# Self-Views and Positive Psychology Constructs Among Second Language Learners in Japan, Taiwan, and the United States

**DOI:** 10.3389/fpsyg.2020.02176

**Published:** 2020-09-11

**Authors:** Xinjie Chen, J. Lake, Amado M. Padilla

**Affiliations:** ^1^Graduate School of Education, Stanford University, Stanford, CA, United States; ^2^Department of English, Fukuoka Jo Gakuin University, Fukuoka, Japan

**Keywords:** cross-cultural study, self-concept, second language learning, motivation, positive psychology

## Abstract

The present study is the first to empirically test a hierarchical, positive-oriented model of the self and its relationship to second language (L2) achievement motivation, and compare it in three different cultural contexts of Japan, the United States, and Taiwan. Based on the L2 self-model (Lake, 2016), three levels of constructs were developed: *Global Self* (i.e., Flourishing, Curiosity, and Hope); *Positive L2 domain self* (i.e., interested-in-L2 self, harmonious passion for L2 learning, and mastery L2 goal orientation); and *L2 Motivational Variables* (i.e., reading, speaking and listening self-efficacy). A total of 667 students participated in this study, including 181 first-year college students in Japan, 159 high school students in Taiwan, and 327 community college students in the United States. All the participants were learning L2 in school. Results showed that the measures of positive global self, L2 domain self, and L2 motivational self all had a stronger relationship within their respective levels, and progressively weaker relationships as level of generality/specificity became more distal. Furthermore, the relationships among measures varied in the differing cultural contexts with the Japan-based student participants relatively lower on all measures. Implications for teacher educators in the L2 context have been discussed.

## Introduction

The field of positive psychology (PosPsy) has grown rapidly in the past decade. While the traditional psychological view focuses on negatives or deficits that need remediation, positive psychology focuses on how people can live optimally and therefore seeks to enable people to grow and reach their full potential (Seligman and Csikszentmihalyi, 2000; Seligman, 1999, 2002, 2011). In psychology, there has been a shared a concern for psychological growth, for example, Maslow (1968) and Rogers (1980), and the humanistic psychology movement have expressed an interest in positive growth. However, much of this work relied on case studies and anecdotal evidence with few empirical studies that would make the psychological constructs more generalizable. Seligman and Csikszentmihalyi (2000) emphasized that positive psychology should be based on empirical data and scientific methods that aim to make research results more replicable and cumulative; the positive focus “does not rely on wishful thinking, faith, self-deception, fads, or hand waving” (p. 7). With this empirical emphasis, the field has incorporated numerous scales to measure positive psychological constructs (e.g., Lopez and Snyder, 2003; Ong and Van Dulmen, 2007), along with bringing older concepts under the positive psychology umbrella.

### Positive Self-Identity in Second Language (L2) Learning

A similar process is taking place within the L2 learning domain as new ideas from positive psychology are combined with previous concepts to create a vibrant subfield with deep roots and a bright future (MacIntyre and Mercer, 2014; MacIntyre et al., 2019). Interest in applying positive psychology to education is a more recent development (White and Murray, 2015). A few researchers have applied positive psychology to the field of L2 learning in a variety of contexts and a range of identity or self-levels from general trait-like to the specific state-like (e.g., Lake, 2013, 2016; MacIntyre and Mercer, 2014; Gabryś-Barker and Gałajda, 2016; MacIntyre et al., 2016; Mercer et al., 2018).

In an article that took a broad look at self-concept and some of the criticisms of it, Swann et al. (2007) argued that self-concepts and outcomes need some sort of contextualization. They point out that broad attitudes and traits were critiqued decades ago for not predicting specific behavior. Now, attitude and trait researchers still use these broad personality constructs with the understanding that there might be mediator or moderator variables in between the broad construct and any particular behavior that determines how predictive of behavior these constructs can be. In the case of self-views, meta-cognitive aspects such as strength of the self-view can bolster predictive validity. Strength of self-view might be indicated by importance, certainty, clarity, extremity, accessibility, temporal stability, or goal-relatedness. To show relationships among variables, researchers need to consider the specificity among the variables with the understanding that there are stronger relationships with variables of similar levels of specificity and less as the relationship in specificity differs (Swann and Bosson, 2010).

### Three-Level Positive Self-Model

**FIGURE 1:**
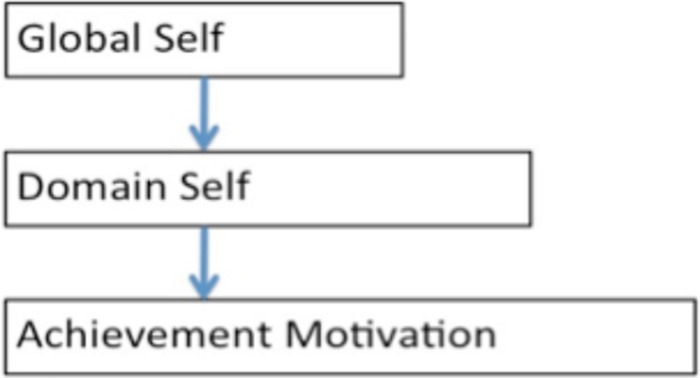
General relationships among levels of self and motivation.

Figure 1 below illustrates in the most abstract case where the structural relations are directed from the global self, to the domain-specific self, to particular motivations. The global level is general in that it relates to the whole self and is relatively stable and trait-like. The middle level is less general and relates to a particular domain or individual interest in life. The domain level relates to a relatively enduring disposition in a particular field or sphere of activity. The achievement motivation level is highly specific to a particular task or set of tasks.

**FIGURE 2:**
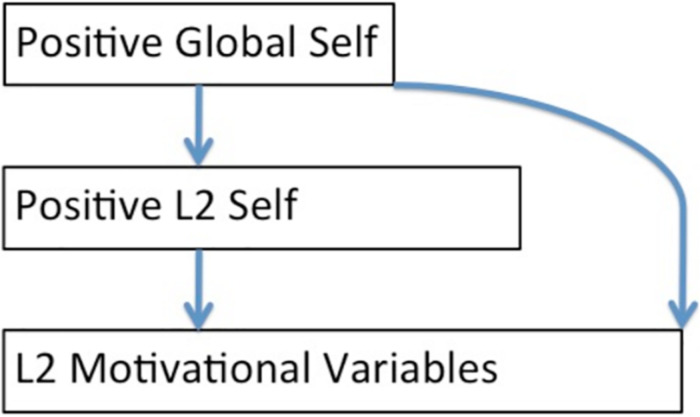
Relationships among levels of positive selves and motivation.

Figure 2 shows an application of the more abstract case of Figure 1. In Figure 2, the structural relations are similarly directed from global, to domain, to particular motivations, but the content is more specific because only the positive dimensions are modeled and a particular domain, L2, is specified.

## Literature Review

Lake (2013, 2016) developed the L2 positive-self model and empirically examined the model constructs and measures in Japan. He found that positive global self-measures, positive L2 self-measures, and L2 self-efficacy measures had stronger relationships not only within a specificity level but also relationships between levels. In the model, *global positive self-constructs* of flourishing, curiosity, and hope, and *positive L2 self-constructs* of interest, passion, and mastery goal orientations are integrated with *L2 self-efficacy* in listening, reading, and speaking. The constructs used were selected within the context of an academic learning environment. Accordingly, flourishing, curiosity, hope, interest, passion, mastery goal orientation, and self-efficacy are situated in the present but are oriented to the future. These differ from constructs such as self-esteem, subjective happiness, positive social relationships, satisfaction in life, and positive and negative trait effects that are situated in the present but are oriented to the past. Although constructs oriented toward the past and the future correlate, the setting of a learning context implies that youthful participants are more oriented toward the future (Lake, 2013, 2014, 2016). Below are the descriptions of the selected variables in this study.

### Positive Global Self

#### Flourishing

Flourishing is a psychological construct that refers to being psychologically healthy. Flourishing is a collection of positive wellness attitudes and behaviors that may persist over time and which signal that a person is prospering psychologically. Flourishing individuals have shown the highest levels of psychosocial functioning in a number of studies (Ryff and Singer, 1998; Keyes, 2002, 2007; Reschly et al., 2008).

Another perspective on flourishing comes from Seligman (2011). Seligman (2002) promoted a version of flourishing where happiness was central. In his 2011 reworking of positive psychology, he advocated for an updated model that focuses on wellbeing composed of five elements: positive emotion (of which happiness and life-satisfaction are aspects), engagement, relationships, meaning, and achievement (PERMA). For Seligman (2011), the target goal of positive psychology is a person who manifests flourishing meaning an individual who is functioning at the highest levels of PERMA.

Some theories of global self are more parsimonious with fewer elements; for example, Deci and Ryan (2000) proposed that differing dimensions of well-being could be subsumed by three basic psychological needs: autonomy, relatedness, and competence. Other theories are more complex and include more elements, for example, Keyes (2007) proposed 13 dimensions that can be loosely grouped into three categories: positive emotions (positive affect and avowed quality of life), positive psychological functioning (self-acceptance, personal growth, purpose in life, environmental mastery, autonomy, and positive relations with others), and positive social functioning (social acceptance, social actualization, social contribution, social coherence, and social integration).

#### Curiosity

Curiosity is a trait-level construct that is not focused on an object or skill and is distinctly different from enjoyment and happiness. That is, like other global self-concepts, the “object” is the self. This makes it clear that the variables in this study are all “subjective” except for the “objective” measure of L2 proficiency. Curiosity refers to “recognizing, embracing, and seeking out knowledge and new experiences” (Kashdan and Silvia, 2009, p. 988). In their study on the development of a curiosity measure, Kashdan et al. (2009) found that curiosity correlated positively with various other positive measures such as openness to experience, happiness, personal growth, autonomy, positive relations with others, and purpose in life. Curious people look for opportunities to acquire knowledge and pursue new experiences. Curiosity helps learners seek and fill in knowledge gaps, recognize potential learning material, and seek new learning situations thus leading to increased achievement and competence (Kashdan and Silvia, 2009).

Curiosity has been shown to have positive relationships to both well-being and learning. Kashdan et al. (2004) suggest that curiosity leads to personal growth through an orientation to stimuli that are novel and challenging, rewarding, and flow-like. In addition, they found measures of curiosity to have a relationship to measures of hope and wellbeing (see also, Kashdan, 2004, 2009). In another study, Kashdan and Yuen (2007) found that when the school environment was supportive of growth and learning, higher levels of curiosity were demonstrated to be associated with higher scores on national achievement exams and school grades. von Stumm et al. (2011) conducted a meta-analysis and found that curiosity had as much influence on academic achievement as intelligence. In brain imaging studies, curiosity has been shown to enhance learning by improving memory by consolidating new information (Kang et al., 2009). Curiosity also activates areas in the brain associated with reward systems so that learning new information can create a stimulus for further learning, that is, “prime a hunger for knowledge” (p. 971).

#### Hope

The hope construct is composed of the elements of clearly defining goals, thinking about ways to achieve those goals, and motivating one to act toward goals. Hope can be characterized and measured as either a trait or state. In this study, hope is measured at the trait level. Hope is composed of two subcomponents that act toward goals, agency or agentic thinking, and pathways or pathway thinking. Agency refers to the belief that one has the ability to initiate, act, persist, and exert effort toward valued goals. It is the belief that one has volition and is in control of making progress toward goals. Sometimes agentic thinking is called willpower. Pathways refer to one’s perceived ability to produce a way or multiple ways to reach a goal, even in the face of obstacles. Sometimes pathway thinking is called *waypower*.

Hope has been associated with wellbeing and learning in a number of studies. Curry et al. (1997) found that hope in college students predicted athletic performance beyond training, academic ability, and global self-worth. Among college students, Chang (1998) found that hope had a positive influence on well-being. Ciarrochi et al. (2007) tested hope, self-esteem, and attribution style for effects on academic achievement and well-being and found that hope had the strongest effect in predicting high school grades and was the only variable to have predictive utility across all outcome measures. Schmid et al. (2011) similarly, found that hope was a better predictor than self-regulatory skills when examining a trajectory of positive youth development.

### Positive L2 Self

As mentioned above, self-concept can refer to different levels of specificity. Components of positive L2 self are composed of L2 domain level dispositional constructs that are positively related to both well-being and second language learning. Constructs at this level are specific to the academic domain or academic language-learning domain, but they are more general than classroom situations and specific language skills and tasks. For the purpose of understanding aspects of the psychology of the language learner relating to positive psychology and learning L2, based on Lake’s (2016) model of positive L2 self, three core aspects of a positive L2 self are elaborated: an interest-in-L2 self, a harmonious passion for L2 learning, and mastery L2 goal orientation.

#### Interested L2 Self

Interested L2 self is short for *an interest-in-L2 self* as a domain-specific mid-level self-concept that can be defined as the disposition to find learning a second language interesting and enjoyable. It is a consequence of believing that one is competent in the L2 and experiencing repeated positive experiences of discovering novel aspects of the language and successfully learning them. The interested L2 self-construct differs from trait-level interest or curiosity, in that trait curiosity does not necessarily have a domain or an object. Interested L2 self also differs from more specific interest states where interest comes first, triggering learning, and then enjoyment comes from having learned. Instead, after frequent instances of state interest and subsequent positive affective states, a more solidified mid-level dispositional interest develops (Silvia, 2006). It is only at the mid-level that interest has a domain and is diffuse enough to overlap with enjoyment and be interpreted as a unitary construct. In other words, feelings of interest and enjoyment at the domain level intertwine. This domain level interest is also similar to the construct of flow, but interested L2 self is a longer-term, more general cognitive and affective dispositional structure that may produce states of flow. Hunter and Csikszentmihalyi (2003) found that for adolescents, there was a strong association between interest and well-being.

#### Harmonious Passion for L2 Learning

The harmonious passion for L2 learning construct has similarities to, but is different from, the interested-in-L2 self. Passion is defined as a strong inclination toward activities that are liked or loved. Where interest theory developed over time from the “bottom-up” based on decades of empirical research, passion theory was created “top-down” from self-determination theory. The model developed by Vallerand et al. (2003); Vallerand (2010) who posited two types of passions, a more self-determined harmonious passion and a more self-uncontrolled obsessive passion. Harmonious passions are associated with adaptive behaviors and obsessive passions with maladaptive behaviors. Passions differ from interests because they trigger activities in which time and energy are spent. Interests might or might not be valued and the time and energy are unspecified. Also, as in self-determination theory, harmonious passions are developed under conditions of autonomy, positive relationships, and competence. Vallerand et al. (2007) found that harmonious passion predicted mastery goals, which, in turn led to deliberate practice and higher performance. Harmonious passion was also found to be related to subjective well-being. In the current study, the passion for L2 learning is short for *harmonious passion* for L2 learning while *obsessive passion* is not part of this study.

#### Mastery L2 Goal Orientation

Mastery goals, which are also known as learning goals, are based on goal orientation theory or achievement goal theory (Dweck and Leggett, 1988; Elliot, 2005) and have to do with building competence. Mastery goals are defined by the purpose or orientation toward absolute gains in learning within an individual. Mastery goal orientation is also called task or learning goal orientation and involves an orientation toward mastery of a task or learning domain (Anderman and Wolters, 2006; Meese et al., 2006). The focus is on learners “concerned with increasing their competence” (Dweck and Leggett, 1988, p. 256). Outcomes are measured as growth from self-comparisons of previous abilities with gained abilities. The second main type of orientation is known as performance goal orientation (also called relative, ego-involved, or competitive goal orientation), in which the focus is on demonstrating competence relative to the competence of others. Outcomes are measured as normative comparisons relative to the abilities of an identified group, such as in a classroom or school. Performance goal orientation is manifest when “individuals are concerned with gaining favorable judgments of their competence” (p. 256). Kaplan and Maehr (1999) found that mastery goal orientations were positively related to well-being measures and academic achievement. Woodrow (2006) found that mastery goal orientations correlated with speaking proficiency as measured by a section of the International English Language Testing System (IELTS). Thus, a mastery goal orientation that is associated with self-improvement, interest, effort, learning, and self-efficacy can contribute toward a positive self. A mastery goal orientation toward learning another language is an aspect of a positive L2 self.

### L2 Self-Efficacy

Bandura (1977) posited self-efficacy as a person’s belief in their ability to succeed, and it has been adopted as a construct in positive psychology (Maddux, 2002; Bandura, 2008). Linnenbrink and Pintrich (2002) note that, “Students who have more positive self-efficacy beliefs (i.e., they believe they can do the task) are more likely to work harder, persist, and eventually achieve at higher levels.” (p. 315). In the field of foreign language learning Hsieh and Schallert (2008) found that among self and differing attribution beliefs, self-efficacy was the best predictor of achievement.

#### Reading Self-Efficacy

Lake (2013) found that L2 reading self-efficacy had positive relationships with positive self-concept variables, positive L2 self -variables, and L2 proficiency. Lake (2014) found that students who read extensively with graded readers gained L2 reading self-efficacy while those who used graded readers, but did not read extensively showed no gains. In addition, gains in L2 reading self-efficacy was shown to have a relationship with gains in a positive reading self as measured by an L2 reading interest measure. In another study with French as a second language, Mills et al. (2006) found reading self-efficacy correlated positively with L2 reading proficiency, in other words, reading proficiency in French could be increased with reading self-efficacy. These few studies show that reading ability is related to an intrinsic factor of motivation and self-efficacy as in L1 reading contexts, but the limited number of studies along with the other studies reviewed support Grabe’s (2009) contention that, “Much more research is needed on L2 reading motivation” (p. 190).

#### Speaking Self-Efficacy

Self-efficacy can be task or domain-specific, that is, it can refer to a particular task that is immediately present or a particular academic domain (Bandura, 1997). When self-efficacy is more general in nature, it becomes similar to the term confidence that is used more colloquially (Bandura, 1997). Lake (2013) found that L2 speaking self-efficacy had positive relationships with positive self-concept variables, positive L2 self-variables, and L2 proficiency.

#### Listening Self-Efficacy

Listening self-efficacy as used here refers to the belief in being capable of successfully listening and understanding at different levels to different sources of spoken language. Lake (2013) found that L2 listening self-efficacy had positive relationships with positive self-concept variables, positive L2 self-variables, and L2 proficiency. Mills et al. (2006) found that listening self-efficacy was associated with listening proficiency only for female, but not male participants in their study.

### Aim of the Research

As mentioned above, Lake (2013, 2016) has examined positive psychology constructs and measures for L2 among Japanese students. He found that positive global self-measures, positive L2 self-measures, and L2 self-efficacy measures had stronger relationships not only within a specificity level but also relationships between levels. The goal of this study was to compare students learning L2 in different cultural contexts to determine whether they react differently to positive psychology constructs. In addition to cross-cultural differences in the interpretation to measures, the relationship between measures may vary according to cultural contexts. Therefore, the aim of the current study is to extend Lake’s positive L2 model to two other cultural contexts—the United States and Taiwan. The research questions seek to explore the similarities and differences across participants in the three areas:

1. What are the relative differences among measures for the participants from the three different cultural contexts—Japan, Taiwan, and the United States?
2. What are the relations between positive self, L2 positive self, and L2 self-efficacy among students in a Japanese, Taiwanese, and American context?
3. If there are differences in the relationships across cultural contexts (Japan, Taiwan, and United States), what form do these differences take and what are the implications for L2 learning?

## Materials and Methods

The measurement instruments used in this study were analyzed using the Rasch rating scale model. The development of specific instruments was described in Lake (2013, 2016).

### Participants

After receiving the approval from the participating schools, students were recruited randomly; those who were enrolled in a foreign/second language class at that time and voluntarily participated in the research were included into our sample. A total of 667 participants were recruited from three different national/cultural backgrounds—Japan, Taiwan, and the United States. Among participants, there were 181 first-year college students in Japan enrolled in English language classes, 159 participants were high school students in Taiwan studying English in afterschool programs, and 327 participants were community college students in the United States.

### Measurement Instruments

In this study, all the measuring scales have been used consistently in the three cultural contexts. In order to avoid any language reading difficulties, the scales were administered in English (in the United States), or translated versions into Chinese (in Taiwan) or Japanese (in Japan). Using a back translation procedure all scales were first translated into Chinese or Japanese and then back-translated to their original language and checked to ensure translation accuracy (Brislin, 1970). All items on the scales were determined to be acceptable. Self-report instruments at three levels of specificity were used to measure components of positive self, positive L2 self, and motivational variables. In this study, a cross-sectional design was used where all scales were administered at the same time with the scale items randomly mixed together into a single questionnaire. All scales were modified or written to have six-item responses that ranged from: *Definitely not true of me; Not true of me; Slightly not true of me; Slightly true of me; True of me; Definitely true of me*.

### Measures for the Global Positive Self

The Curiosity and Exploration Inventory II (CEI-II; Kashdan et al., 2009) is a scale designed to measure trait curiosity. The CEI-II contains five items measuring a dimension of curiosity about seeking new knowledge and experiences (e.g., *I actively seek as much information as I can in new situations*) and five items that measure a dimension of curiosity about a general willingness to embrace the novel, uncertain and unpredictable in life (e.g., *I am the type of person who really enjoys the uncertainty of everyday life*). Kashdan et al. (2009) reported alpha reliabilities of 0.85 and 0.86. They also suggested that because the two dimensions strongly correlate (*r* = 0.79), the 10 items might be used together as a single scale. The alpha reliability for the present study was 0.90.

The Hope scale (Snyder et al., 1991; Snyder, 2000) is an eight-item scale that measures trait-level hope. The hope construct consists of two factors. Four items reflect agentic thinking about one’s goals (e.g., *I meet the goals that I set for myself*) and four items reflect a pathways thinking about the ways to achieve goals (e.g., *There are a lot of ways around a problem*). Reported alpha reliabilities have ranged from 0.74 to 0.88. The alpha reliability for the present study of the combined measure of hope (agency) and hope (pathways) was 0.90.

The Flourishing scale (Diener et al., 2010) consists of eight items describing aspects of positive functioning and human flourishing (e.g., *I actively contribute to the happiness and well-being of others*). The alpha reliability reported was 0.87, and for the present study, it was 0.91.

### Measures for the Positive L2 Self

For the Interested L2 Self scale, seven adapted items from previous studies were used (e.g., *English is an interesting field of stud*y (Lake, 2013, 2016). The reliability from previous studies was 0.91 and 0.92 (Lake, 2013, 2016). The alpha reliability for the measure in the present study was 0.93.

For the L2 Mastery Goal scale, seven adapted items from previous studies were used (e.g., *I like learning difficult things in this class*; Lake, 2013, 2016). The reliability from previous studies was 0.87 and 0.94. For the seven items in the present study, the alpha coefficient was 0.94.

Lake (2013, 2016) adapted items (e.g., *I am passionate about learning English*) from the Harmonious Passion subscale (Vallerand et al., 2003) to create a Passion for L2 scale that he used with students in Japan learning English. The scale reliability in previous studies was 0.90 and 0.90 (Lake, 2013, 2016). The scale used in this study with the Japan sample consisted of seven items; however, the scale used with the Taiwan and United States group had six items. Five of the items were similar to all three groups. The alpha reliability for the present study was 0.79.

### Measures of L2 Self-Efficacy

The L2 self-efficacy variables are specific to L2 learning, skills, and tasks. Variables at this level are more dynamic or less trait-like because of their specificity and the situational nature of the contexts, processes, and specific tasks. The self-efficacy items were taken from previous studies (Lake, 2013, 2016).

The Speaking Self-Efficacy scale consisted of nine items that were adapted from previous studies (e.g., *I can give a speech in English*). The reliability was 0.90 in an earlier study (Lake, 2016). The alpha reliability for the present study was 0.96. The Listening Self-Efficacy measure used nine adapted items in this study (e.g., *I can understand the main ideas when listening to English songs*); reliability was 0.89 (Lake, 2016). For the present study reliability was 0.96. The Reading Self-Efficacy measure used seven adapted items in this study (e.g., *I can read and understand a menu in English*). The alpha reliability was 0.89. For the present study, alpha was 0.97.

### Procedures

Students in Japan were given a paper-pencil survey questionnaire, while students in Taiwan and the United States completed an online Qualtrics survey during or after class time. The different modality of instrument used in each area was based on the available access method provided by the participating schools. On average, participants took approximately 20–25 min to complete the questionnaire. Prior to distributing the survey, our research was approved by the review committee of the university. All participants were informed about the nature of the study and told that participation was voluntary. Participants have the right to stop at any time. Rasch analysis was done to get measures in logits for each student and then to examine the relationships.

The analysis was done in two steps. First, the measures were analyzed with the total participants (*n* = 667). This was done to get the relative mean and standard deviation statistics. Second, the measures were analyzed by cultural context to determine relationships among measures within each participant group.

## Results

Descriptive statistics for the measures in logits and alpha reliabilities are presented in Table 1. The items and scale measures met the assumptions of the Rasch rating scale model for a well-formed scale. In other words, item fit statistics were acceptable, average measures advanced monotonically with categories, step calibrations or thresholds advanced with appropriate higher values, and no additional dimensions to each measure were found to suggest violations of unidimensionality. For more information about measure construction and Rasch rating scale development, please see literature (e.g., Bond and Fox, 2015; Boone et al., 2014; Engelhard and Wind, 2018).

**TABLE 1:** Descriptive Statistics of measures in logits used in this study.

Measures	M	SD	Alpha
Curiosity	0.70	1.30	0.90
Flourishing	1.16	1.67	0.91
Hope	1.25	1.62	0.90
Interested in L2 Self	1.87	2.40	0.93
Mastery Goal Orientation	2.21	2.64	0.94
L2 Harmonious Passion	1.62	2.26	0.79
L2 Reading Self-efficacy	1.07	3.62	0.97
L2 Listening Self-efficacy	1.09	3.25	0.96
L2 Speaking Self-efficacy	0.88	2.95	0.96

*n = 667.*

In the next step of the analysis, logits were computed for all measures for each of the three cultural groups in the study. Table 2 shows the relative values for each measure, in logits, by cultural context. Correlations were next estimated for each measure by three cultural contexts (area groups).

**TABLE 2:** Mean and standard deviations for each measure in logits by cultural context.

Measures	M	SD
Curiosity		
Japan	–0.09	1.14
Taiwan	1.23	1.19
United States	0.88	1.23
Flourishing		
Japan	–0.29	0.96
Taiwan	1.54	1.54
United States	1.78	1.55
Hope		
Japan	0.01	1.43
Taiwan	1.75	1.25
United States	1.69	1.51
Interested in L2 Self		
Japan	1.38	2.52
Taiwan	1.95	2.37
United States	2.10	2.32
Mastery Goal Orientation		
Japan	0.34	2.37
Taiwan	3.11	2.39
United States	2.80	2.39
L2 Harmonious Passion		
Japan	0.44	2.05
Taiwan	1.95	2.19
United States	2.12	2.18
L2 Reading Self-efficacy		
Japan	–1.50	2.03
Taiwan	3.04	2.89
United States	1.54	3.83
L2 Listening Self-efficacy		
Japan	–1.42	1.50
Taiwan	2.28	2.96
United States	1.90	3.36
L2 Speaking Self-efficacy		
Japan	–1.27	1.53
Taiwan	1.79	2.81
United States	1.63	3.02

*Japan *N* = 181; Taiwan *N* = 159; United States *N* = 327.*

The results of these analyses were shown in Table 3.

**TABLE 3:** Correlations of measures by cultural context.

Measures	Context	1	2	3	4	5	6	7	8	9
					**Positive Self Measures**					
(1) Curiosity	Japan	1								
	Taiwan	1								
	United States	1								
(2) Flourishing	Japan	0.73	1							
	Taiwan	0.43	1							
	United States	0.54	1							
(3) Hope	Japan	0.82	0.86	1						
	Taiwan	0.61	0.67	1						
	United States	0.59	0.73	1						
					**Positive L2 Self Measures**					
(4) Interested in L2	Japan	0.60	0.54	0.58	1					
	Taiwan	0.26	0.33	0.32	1					
	United States	0.35	0.32	0.28	1					
(5) Mastery Goal	Japan	0.67	0.61	0.63	0.88	1				
Orientation	Taiwan	0.43	0.45	0.45	0.71	1				
	United States	0.45	0.47	0.42	0.70	1				
(6) L2 Passion	Japan	0.62	0.57	0.59	0.88	0.90	1			
	Taiwan	0.30	0.44	0.45	0.72	0.66	1			
	United States	0.35	0.34	0.27	0.73	0.70	1			
					**L2 Motivation Measures**					
(7) L2 Listening	Japan	0.55	0.52	0.52	0.62	0.68	0.68	1		
Self-efficacy	Taiwan	0.21	0.20	0.20	0.16	0.11	0.29	1		
	United States	0.16	0.11	0.16	0.14	0.25	0.27	1		
(8) L2 Reading	Japan	0.52	0.53	0.51	0.61	0.68	0.66	0.83	1	
Self-efficacy	Taiwan	0.23	0.23	0.19	0.19	0.08	0.08	0.88	1	
	United States	0.15	0.05	0.15	0.15	0.23	0.24	0.78	1	
(9) L2 Speaking	Japan	0.59	0.64	0.61	0.59	0.70	0.64	0.83	0.83	1
Self-efficacy	Taiwan	0.22	0.19	0.17	0.12	0.10	0.10	0.91	0.86	1
	United States	0.15	0.10	0.17	0.18	0.28	0.30	0.88	0.81	1

*Japan N = 181, Taiwan N = = 159, United States N = 327.*

Finally, in Table 4, correlations were calculated to show average relationships within and between levels for each cultural (area) group. The average correlations within levels are in the diagonal, and those between levels are below the diagonal.

**TABLE 4:** Average correlations within and between levels.

Average correlations		Positive self measures	Positive L2 self measures	L2 motivation measures
Positive Self Measures				
	Japan	0.80		
	Taiwan	0.57		
	United States	0.62		
Positive L2 Self Measures				
	Japan	0.60	0.89	
	Taiwan	0.38	0.70	
	United States	0.36	0.71	
L2 Motivation Measure				
	Japan	0.55	0.65	0.83
	Taiwan	0.20	0.14	0.88
	United States	0.13	0.23	0.82

## Discussion and Implications

Looking at the measures analyzed show that each measure had a high alpha reliability (+0.90) except for the L2 harmonious passion measure (0.79), which while still within the acceptable range, is lower than the other variable. Due to an error in administering the items, the Japan group did not include one item included in the other two groups and the Taiwan and United States groups did not include two items included in the Japan group. The passion measure in this study then had five items in common which acted as an anchor with the omitted items treated as missing in the Rasch rating scale analysis for the combined groups. This caused no major adverse effects except for perhaps lowering the reliability slightly. However, at 0.79, the reliability for the combined groups is acceptable.

An examination of the means in differing cultural contexts in Table 2 demonstrates that the Japanese group was lower on all measures. Possibly this suggests that the Japanese participants do not “self-enhance” (Leary, 2007) or under-report or “self-verify” (Swann et al., 2007) on self-report measures compared to United States-based participants. However, the Taiwan group did not follow the same pattern suggesting that this is not simply an East-West dichotomy, but that perhaps other situational factors are coming into play. The Taiwan group with high school participants in after-school voluntary English classes, the Japan group with university students in mandatory English classes, and the United States group of young college adults enrolled in second/foreign language classes might have contributed to the effects of age or L2 interest.

Averages of intercorrelations in Tables 3, 4 show generally stronger intercorrelations in the Japanese group. This needs to be studied further, but we believe that possibly the Japanese students had developed a more mature understanding and commitment to learning L2 because of their instrumental interest in the subject matter of English or because of their interest in international careers. Thus, the Japanese students, because of their university status, may have developed to a higher level of self than the younger Taiwanese participants and the United States students enrolled in L2 classes. In other words, the Japanese students may have a more integrated sense of their positive “selves” because of a commitment to the L2 as an integral aspect of their career aspirations which demand knowledge of English, the language they were studying at the time of completing the survey.

Although the strength of the intercorrelations varied among groups as seen in Tables 3, 4, patterns within and between levels were the same for all groups. In other words, in the diagonal, the intercorrelations or averages of intercorrelations demonstrate that within a level, measures correlate more highly than with other levels. For example, within the global positive self-level, curiosity, flourishing, and hope are positively correlated with each other. This result is consistent with previous literature that indicates that individuals who score higher on flourishing tend to have a higher sense of hope and are more curious (Gunderman, 2008) than individuals who are lower on the measure of flourishing. Conversely, with greater curiosity and hope, people are more likely to flourish and express a higher level of well-being (Keyes, 2007). This can be explained by the broaden-and-build theory (Fredrickson, 1998) that states that individuals who tend to be curious are more open to new experiences, possess more receptive attitudes toward new ideas, and accumulate new knowledge that adds to their personal resources (e.g., psychologically, cognitively), which enables them to cope better with uncertainty and more likely to flourish (Kashdan, 2004).

In addition, the global positive self-measures (i.e., curiosity, flourishing and hope) correlated with the L2 domain self-measures (i.e., interested in L2, mastery goal, and L2 passion) more strongly than the positive L2 self-efficacy measures (i.e., speaking, listening, and writing self-efficacy). The positive L2 domain self-measures in turn generally correlated more highly than the positive global measures with the L2 self-efficacy measures. The exception was for the Taiwan group with a very small (0.06) difference between the positive self-measures (*r* = 0.20) and L2 positive self-measures (*r* = 0.14). Overall, these results are consistent with previous literature. In other words, students scoring higher on the positive global self are also higher in the L2 self and on the L2 motivational variables (Lake, 2013). Furthermore, these results support the “specificity matching principle” (Swann et al., 2007, p. 87) showing that relationships among self-constructs will be stronger within a level of specificity and will weaken as levels of specificity become more distal. The difference across the three cultural groups could be due to a less differentiated self among the younger Taiwanese students.

The findings reported in this study have practical implications for L2 educators. We showed that a significant relationship exists between the positive self and L2 self among students who vary by age, reasons for studying L2, and across three different cultural and linguistic contexts (i.e., Japan, Taiwan, and the United States). These findings support the applicability of Lake’s (2013) theoretical model that argues that there are beneficial effects to be derived through the use of strategies for enabling L2 learners to increase their curiosity and hope that in studying L2 they can achieve their desired goal of becoming proficient in the L2. This supports a general self in L2 learning motivation through building positive self-concepts specific to the domain of L2 learning (e.g., interest in L2, passion, and mastery goal orientation). In addition, since the patterns within and between levels were the same for participants in all three groups, this suggests that generally these intercorrelational effects are applicable among L2 learners regardless of differences in cultural and language contexts. Accordingly, we recommend that L2 educators would do well in their instructional practices to promote students’ global well-being by creating positive L2 learning environments, such as setting up challenging but attainable goals that are matched to students’ ability. Teaching L2 is more than just focusing on specific linguistic outcomes but is dependent also on aligning instruction with students’ interests and using more mastery-oriented verbal compliments related to effort and persistence in class activities. The goal of such instruction is to promote students’ curiosity, passion, and interest in L2 learning and, thereby, enhancing learners’ sense of self-efficacy while studying the L2.

## Conclusion and Limitations

In sum, this study examined positive psychological self-constructs at three levels of generality, global self, and L2 domain self, and L2 motivational self-levels based on studies by Lake (2013, 2016). It was found that the Japan-based group was lower on all measures relative to the Taiwanese or United States comparison groups. The relationships among measures varied to a degree in the differing cultural contexts. However, it was found that the general pattern of relationships was similar, that is, positive global self, L2 domain self, and L2 motivational self-measures all had a stronger relationship within the levels and progressively weaker relationships as the level of generality/specificity became more distal. In addition, this study suggests that rather than broad cultural comparisons, it may be more meaningful to examine smaller cultural or situational contexts that may influence students differently (Holliday, 1999). This sheds light on the importance of analyzing the learning of L2 issues at a deeper level, instead of purely from the traditional lens of an East and West dichotomy. In addition to the broad cultural variables such as mandatory versus elective language (L2) classes, required language classes for field of interest versus non-major language classes, and age or developmental differences may play a greater role in how a student construes his/her self. Several possible limitations should also be recognized. First, the numbers of participants between Taiwan, United States, and Japan are not balanced. While the question of unequal sample sizes is not uncommon in comparative research such as this, it bears mentioning. Second, because of constraints of data collection across geographic areas, some potential factors of importance could not be considered such as language proficiency, level of motivation, specific learning contexts, and exposure to native English speakers. Third, the study relied on self-reports for all the measures across all national groups. In the future, more measurement tools need to be identified that do not rely exclusively on self-reports. For example, triangulating the data through interviews would shed more light on the richness of the results. Future studies might investigate some of the limitations of this study.

## Data Availability Statement

The datasets presented in this article are not readily available because confidential data. Requests to access the datasets should be directed to xjchen96@stanford.edu.

## Ethics Statement

The studies involving human participants were reviewed and approved by University Human Subjects (IRB) – Research Compliance Office. Written informed consent to participate in this study was provided by the participants’ legal guardian/next of kin.

## Author Contributions

All authors listed have made a substantial, direct and intellectual contribution to the work, and approved it for publication.

## Conflict of Interest

The authors declare that the research was conducted in the absence of any commercial or financial relationships that could be construed as a potential conflict of interest.
